# Prolyl-glycyl-proline (PGP) Peptide Prevents an Increase in Vascular Permeability in Inflammation

**Published:** 2017

**Authors:** N. S. Bondarenko, A. N. Shneiderman, A. A. Guseva, B. A. Umarova

**Affiliations:** Koltsov Institute of Developmental Biology, Vavilova str. 26, Moscow, 119334, Russia; Institute of Carcinogenesis, Cancer Research Center of N.N. Blokhin, Kashirskoe sh. 24, Moscow, 115478 , Russia; Lomonosov Moscow State University, Leninskie Gory 1, Moscow, 119991, Russia

**Keywords:** anti-inflammatory action, inflammation, prolyl-glycyl-proline (PGP), vascular permeability

## Abstract

This study was aimed at investigating the effect of prolyl-glycyl-proline (PGP) tripeptide on vascular permeability in rats with an inflammation. It was found that the peptide reduces the rat paw edema induced by a subcutaneous administration of histamine to the same extent as the conventional anti-inflammatory agent diclofenac. However, an assessment of the relative expression level of the *cox-2 *gene at the inflammation focus using real-time PCR showed that, in contrast to diclofenac, PGP does not affect the *cox-2 *gene expression. This is indicative of the fact that they have different mechanisms of action. We used the model of acute peritonitis induced by an intraperitoneal injection of thioglycolate to demonstrate that the inflammatory response of an organism is accompanied by increased vascular permeability in the tissues of the stomach and small intestine. Pre-administration (30 minutes before the induction of the inflammation) of PGP prevented this increase, whereby the level of vascular permeability, exudate volume in the peritoneal cavity, and the amount of the Evans Blue dye in this exudate remained at the control level. Therefore, these results suggest that the anti-inflammatory action of PGP is based on its ability to prevent an increase in vascular permeability.

## INTRODUCTION

Most pathological processes in the body are accompanied by the development of an inflammatory response. It is important to understand the role of endogenous regulatory systems in this process in order to restore the disturbed functions of the organism. Regulatory peptides that are formed in the inflammation focus as a result of a degradation of extracellular matrix proteins are of great interest. Prolyl-glycyl-proline (PGP) tripeptide, a typical representative of the glyprolines family, is one of such peptides [1]. To date, a wide range of properties attributed to this peptide that are indicative of its protective effect on the inflammation-related disorders of various body systems have been discovered. In particular, PGP and its metabolites have a protective and therapeutic effect on the gastric mucosa in ulcerogenesis of various etiologies [2] and reduce the number of neutrophils in the inflammation focus in rats with peritonitis induced by intraperitoneal injection of a 1% acetic acid solution [3]. In an experimental model of acute peritonitis (intraperitoneal injection of a 40% thioglycolate solution), we demonstrated that the peptide has a pronounced anti-inflammatory effect to a certain degree mediated by mast cell stabilization [4, 5]. Mast cells are a source of many vasoactive mediators which increase vascular endothelial permeability [6], one of the first signs of an inflammatory response [7]. Based on this fact, we assumed that the PGP can affect the vascular permeability and, consequently, formation of edema in the inflammatory response. Our study focuses on an investigation of this issue.

## EXPERIMENTAL

PGP peptide was synthesized at the Institute of Molecular Genetics, Russian Academy of Sciences, Moscow.

In this study, we used male albino rats weighing 180–220 g. The experiments complied with the ethical principles and regulations as recommended by the European Science Foundation (ESF) and the Declaration on the Humane Treatment of Animals.

A rat paw edema was induced by subcutaneous injection of 0.2 mg of histamine (Sigma, USA) in 0.1 ml of saline. The thickness and circumference of the paw was measured every hour for 4 hours.

Two hours after inflammation induction with histamine, the animals underwent excision of a tissue sample from the inflammation focus under ether anesthesia; the sample was immediately placed into a RNAlater solution (Ambion).

RNA from the tissue samples was isolated using TriReagent (Sigma, USA) according to the manufacturer’s protocol.

Single-stranded cDNA was obtained by adding 80 pmol of random 9-mer primers to 2 μg of the pooled RNA pretreated with DNAse I (Fermentas, USA) (1 unit of enzyme per 1 μg of RNA) and incubation for 5 min at 70°C. Then, 25 μl of RT-MIX (RT-Buf 10 × (Fermentas, USA)), 40 mM of dNTP, and 10 units of RNAsin (Promega, USA) were added to the ice-cooled mixture and incubated for 5 min at 37°C. Further, 200 units of M-MuLV reverse transcriptase (Fermentas, USA) was added and reacted at 42°C for 1 hour. The reaction was stopped by heating the mixture at 70°C for 10 min. The sample was diluted to 200 μl with deionized water. Samples were stored at –20°C.

Real-time PCR was performed using an iCycler iQ5 thermocycler (BioRad Laboratories GmbH, Germany). cDNA was used as a PCR template. The reaction mixture contained 10 pmol of the 5’- and 3’-primers, 1.6 mM of MgCl_2_, 0.25 mM of dNTP, 10 × amplification buffer with the intercalating fluorescent dye Eva Green (Synthol, Russia), and 1 unit of Taq-DNA polymerase (Synthol, Russia). The reaction mixture was pre-heated for 10 minutes at 95°C and then subjected to 40 cycles of denaturation (95°C), annealing (60°C), and polymerization (72°C), accompanied by an assessment of the amount of accumulated product using the fluorescence spectrum at the end of the elongation stage. Each reaction was performed in triplicates. We used the following primers: *cox-2 *F–5’-CCATGTCAAAACCGTGGTGAATG-3’, *cox-2 *R–5’-ATGGGAGTTGGGCAGTCATCAG-3’, *gapdh *RN F–5’-CTGACATGCCGCCTGGAGAAA-3’, *gapdh *RN R–5’-TGGAAGAATGGGAGTTGCTGTTGA-3’.

The housekeeping gene *Gapdh *was used as a reference to adjust the amount of transcripts added to the reaction. The primers were selected using the VectorNTI program. The primer annealing temperature and the number of amplification cycles for each fragment were selected experimentally. Experimental results were processed, and the relative expression level was calculated using Bio-Rad iQ5 2.0 Standard Edition Opticaland and the REST 2005 software. The reaction efficiency coefficient was calculated using the LinRegPCR program.

Acute experimental peritonitis was induced in the rats by intraperitoneal injection of a 40% thioglycolate solution (Fluka, Switzerland) at a dose of 4 g/kg, followed by measurement of the vascular permeability in the stomach and small intestine. For this purpose, an Evans blue dye (Sigma, USA) was injected (50 mg/kg) into the jugular vein using a syringe. In 15 minutes, the animals were anesthetized, dissected, and fluid samples were collected. The animals were then transcardially perfused for 2–3 minutes with saline containing 20 units/ml heparin. The stomach and small intestine were isolated, fragmented, and weighed. The dye was extracted by soaking tissues in formamide (Scharlau Chemie SA) for 48 hours at 37°C. The samples were then centrifuged for 15 min at 2000 rpm. The amount of the dye was determined using a Multiscan EX spectrophotometer (Thermo Scientific) at λ = 620 nm and expressed in μg/g of tissue and in μg/ml of exudate.

The results were processed statistically using the nonparametric Mann-Whitney test in the STATISTICA 6 software. Threshold significance value was set to 0.05.

## RESULTS AND DISCUSSION

**Fig. 1:**
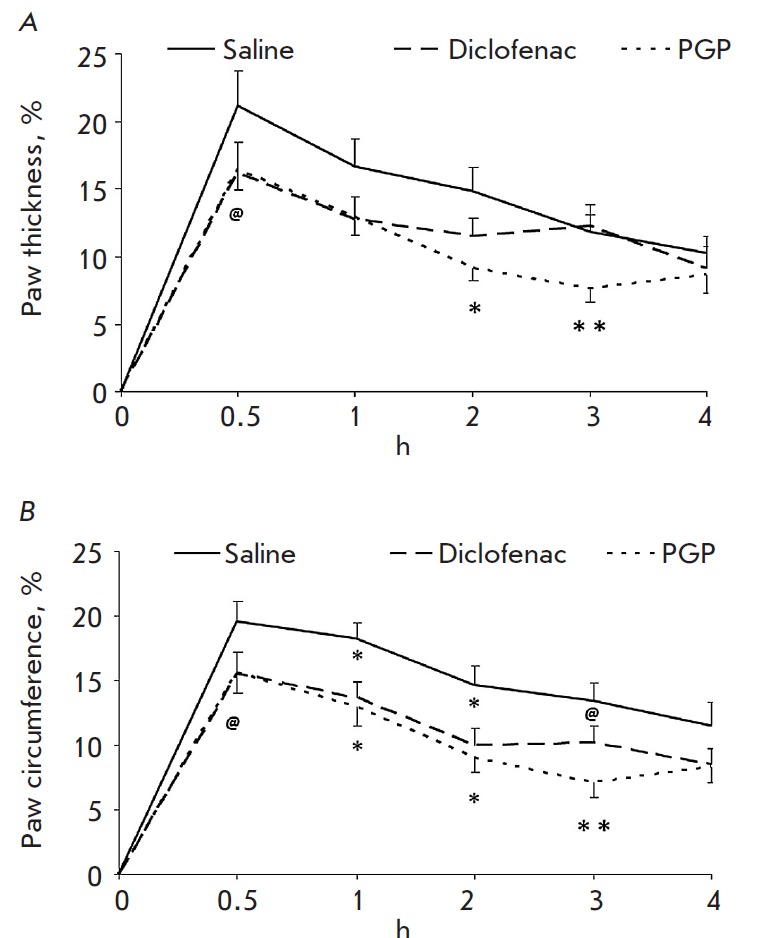
The effect of PGP and diclofenac on the change in the thickness (A) and the circumference (B) of a rat paw in a histamine-induced inflammation. * – p < 0.05, ** – p < 0.01, @ – p < 0.1.

In the first series of experiments, the animals were subcutaneously injected with 0.2 mg histamine in a paw at a volume of 100 microliters in order to induce the edema. The same paw was intramuscularly injected with PGP at a dose of 3.7 μmol/kg 30 min before the induction of the inflammation. Another group of experimental animals received diclofenac at a dose of 1 mg/kg (Hemopharm, Serbia). Control animals were injected with saline instead of peptide. The thickness and circumference of the paw were measured and expressed as a percentage with respect to the baseline (before drug administration). The results are shown in *Fig. 1*.

The thickness and circumference of the paw 30 minutes after administration of histamine increased on average by 20.5%. After pretreatment with peptide, paw swelling was significantly less pronounced. As early as in 1 hour, paw circumference decreased by 29.5% compared to the control; in 2 and 3 hours – by 38 and 47%, respectively. Two hours after induction of the inflammation, paw thickness was 38% lower than in the control; in 3 hours – 35% lower.

**Fig. 2:**
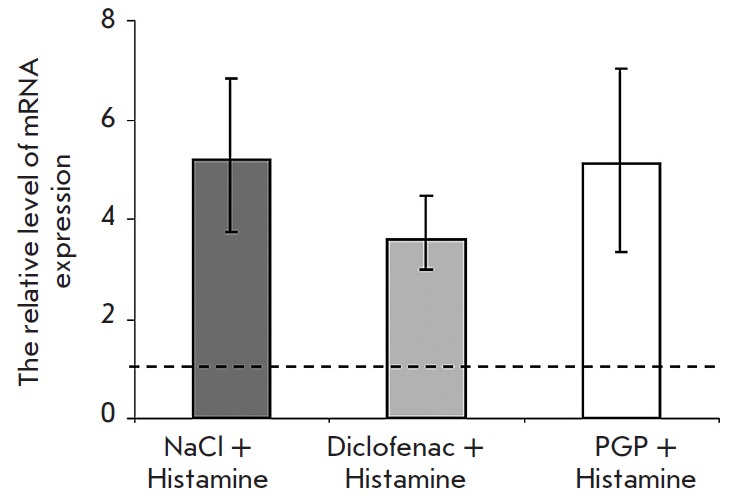
The level of COX-2 mRNA expression. The dotted line shows the normal expression level.

Thus, pre-treatment with PGP resulted in a reduced edema. This confirms the previously discovered anti-inflammatory effect of the peptide. When comparing the effects of PGP and NSAID diclofenac (*Fig. 1*), it was found that the peptide and diclofenac are characterized by a similar action. They both reduced the edema induced by histamine. This suggests a possible similarity in their mechanisms of action. It is known that diclofenac inhibits cyclooxygenase-2 (COX-2), one of the key enzymes in the chain of prostaglandin synthesis from arachidonic acid. For this reason, in the next series of experiments, we investigated the effect of PGP on the *cox-2 *gene expression during an inflammation caused by subcutaneous administration of histamine in the paw. The results are shown in *Fig. 2*.

Subcutaneous injection of histamine resulted in an almost fivefold increase in *cox-2 gene *expression 2 hours after the inflammation onset*. *Pretreatment with diclofenac decreased the level of expression of the investigated gene by 31%. However, injection of the peptide did not alter the level of gene expression and it remained the same as in the inflammation. This reflects differences in the antiedematous mechanisms of PGP and diclofenac.

Since the formation of an edema is caused by an increased permeability of the vascular endothelium, we evaluated the effect of PGP on the change in vascular permeability in the stomach and small intestine in rats with acute peritonitis.

The study group animals received an intramuscular injection of PGP at a dose of 3.7 μmol/kg 15 minutes before an intraperitoneal injection of thioglycolate. In the other group, the animals received saline instead of PGP. In the third group, the animals received saline without a subsequent induction of inflammation. The level of vascular permeability was assessed based on the amount of Evans blue dye in the exudate and tissue samples from the stomach and intestine.

**Fig. 3:**
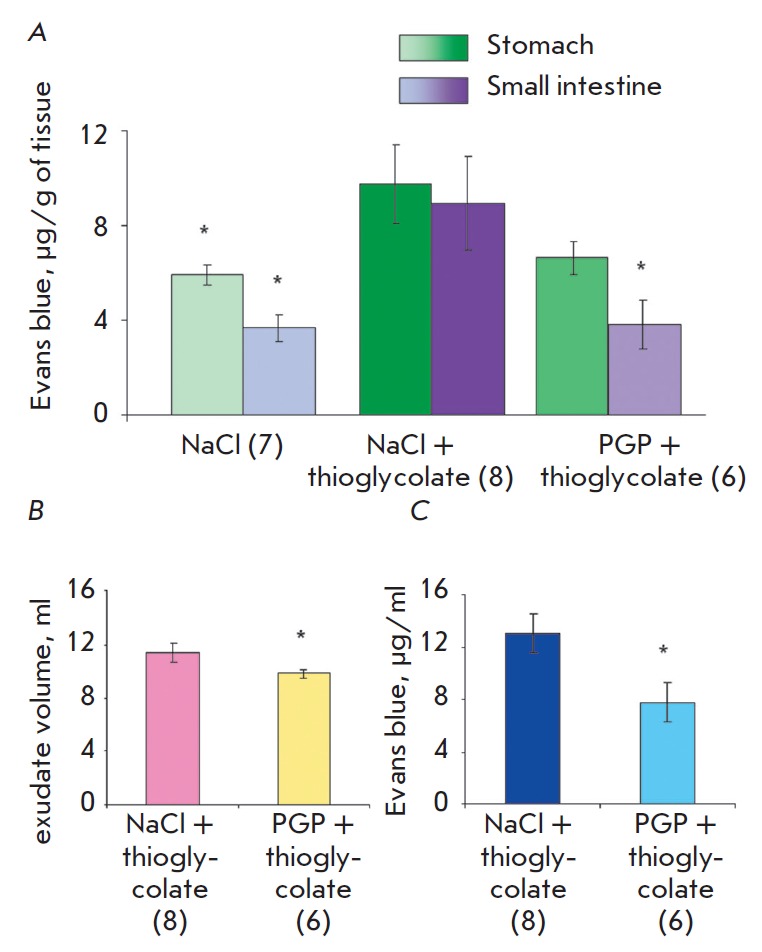
Changes in the vascular permeability in the stomach and small intestine (A), peritoneal exudate volume (B), and the amount of Evans blue dye in the exudate (C) in rats with experimental acute peritonitis 30 minutes after thioglycolate administration. * – p < 0.05. The numbers in parentheses indicate the number of animals in each group.

The results are shown in *Fig. 3*. It is clear that the vascular permeability in the control (5.9 μg of dye per 1 g in the gastric tissue and 3.7 μg/g in the intestinal tissue *(Fig. 3A)) *differs significantly from the vascular permeability during inflammation. Vascular permeability in the stomach increased on average by 66%, while in the intestines it increased 2.4-fold. Pre-administration of the peptide prevented an increase in vascular permeability in the stomach and intestines. Increased permeability after the administration of thioglycolate and the effect of peptide administration persisted for 2 hours.

Furthermore, the decrease in the vascular permeability is evidenced by the reduced amount of exudate in the peritoneal cavity by 14% and an almost twofold lower amount of the dye in the exudate (μg/ml) *(Fig. 3 B,C*).

Therefore, pre-administration of PGP prevents an increase in vascular permeability in the stomach and small intestine. The exudate volume and the amount of the dye in it also remain at the control level.

The ability of PGP to prevent an increase in the vascular endothelial permeability may be associated with both the direct action of the peptide on blood vessels [8] and its stabilizing effect on the mast cells [9], whose mediators are known to contribute much to the initiation and regulation of the vascular permeability. During our experiments, we found that the peptide cannot be considered as a conventional anti-inflammatory drug such as diclofenac, since, despite the formal similarity of the observed effects, the mechanisms of action are different.

The data on the participation of PGP in the regulation of the inflammatory process are contradictory. On the one hand, the protective effect of glyprolines on various inflammation-related disorders was conclusively established. Thus, they not only have a protective effect on the gastric mucosa during the formation of ulcers, but also protect cells from oxidative stress and are involved in the regulation of the immune status of the organism [10].

On the other hand, PGP and its N-acetylated derivative (N-AcPGP) are chemoattractants for neutrophils [11]. This activity is mediated by an interaction with CXCR2 neutrophil receptors. These peptides attract neutrophils to the inflammation focus and thus prolong the process. However, CXCR2 receptors are ligand-selective, which can trigger signaling cascades both enhancing and limiting inflammation [12]. Furthermore, there is recent evidence showing that binding of N-AcPGP to the CXCR2 receptors of neutrophils, monocytes, and macrophages contributes to the therapeutic effect of this peptide in sepsis. It was found that administration of N-AcPGP to mice results in increased production of interferon-γ and IL-12, as well as an inhibition of the production of proinflammatory cytokines (TNF-α, IL-1β, and IL-6) in the peritoneal fluid. N-AcPGP directly increased the *in-vitro *production of interferon-γ and decreased the lipopolysaccharide-stimulated production of TNF-α by murine spleen macrophages and human leukocytes [12]. These data indicate that the chemoattractant activity of the peptides does not preclude an ability to exhibit anti-inflammatory properties under certain conditions.

## CONCLUSION

Our collection of data suggests that the anti-inflammatory action of the peptide is based on both a mast cell stabilization and its ability to prevent an increase in vascular permeability.
